# 229 Evaluating False-Positive Norovirus Results on Multiplex GI PCR Testing in Clinical Practice

**DOI:** 10.1017/ash.2026.10608

**Published:** 2026-06-23

**Authors:** Carrie Billman, Lindsay Bow, Sara Pau

**Affiliations:** 1 The Johns Hopkins Hospital; 2 Johns Hopkins Hospital

## Abstract

**Background:** In response to the 2014 Ebola outbreak, Regional Emerging Special Pathogen Treatment Centers (RESPTCs) were established across the 10 U.S. Health and Human Services regions to maintain a high-level of preparedness to care for patients with high consequence infectious diseases (HCID) in specially designed biocontainment units (BCU). The Johns Hopkins BCU purposefully integrated infection prevention and control principles and practitioners into operational plans as the foundation of healthcare worker safety in the high-level isolation environment. Infection preventionists (IPs) serve as Trained Observers (TOs) who are responsible for oversight of enhanced personal protective equipment (PPE) donning and doffing procedures and overall safety throughout the clinical shift. The TO role is fulfilled exclusively by IPs who are essential to maintaining BCU staff proficiency by facilitating ongoing quarterly training. **Methods:** A comprehensive training and validation framework was implemented to sustainably embed IPs as TOs into the BCU program. First, a dedicated IP BCU program manager role was established to integrate infection prevention into ongoing protocol development and operational decision-making. Next, TO candidates underwent a standardized competency-based process that required demonstration of proficiency in leading enhanced PPE ensemble donning and doffing procedures. Additionally, TO candidates were expected to confidently demonstrate effective use of structured checklists and standard operating procedures, navigate BCU workflow and infrastructure, and be familiar with Category A waste management protocols. Risk identification and mitigation skills were reinforced through peer coaching and drilled over the course of multiple practice and training sessions. TO validation required independent confirmation of candidate proficiency by two validated TOs to ensure inter-rater consistency. **Results:** Over 11 years, 23 IPs achieved TO validation and continued to revalidate and maintain their skill set throughout their IP tenure during BCU quarterly training. Currently, 8 of 17 IPs are active TOs. IP integration into BCU training and operations fosters increased trust and a heightened sense of safety across the team. Dedicated TOs with the sole responsibility to ensure healthcare worker safety allows for deeper specialization and improved adherence to safety standards, unburdening other clinicians from balancing multiple roles between bedside and operational safety. **Conclusion:** Embedding IPs as TOs within RESPTC preparedness plans and BCU response operations—beyond their traditional role in developing standard operating procedures, processes, and protocols—is a best practice to promote a culture of safety and an innovative approach to support operational efficiency for patient care in high-level isolation environments.

